# Coinfection with Canine Distemper Virus and Yellow Fever Virus in a Neotropical Primate in Brazil

**DOI:** 10.3390/v16111670

**Published:** 2024-10-25

**Authors:** Maria Angélica Monteiro de Mello Mares-Guia, Marina Carvalho Furtado, Flávia Löwen Levy Chalhoub, Maria Dulce Portugal, Janice Mery Chicarino de Oliveira Coelho, Ana Maria Bispo de Filippis, Felipe Gomes Naveca

**Affiliations:** 1 Arbovirus and Hemorrhagic Virus Laboratory, Oswaldo Cruz Institute, Rio de Janeiro 21040-360, Brazil; flaviallevy@yahoo.com.br; 2 Anatomic Pathology Service, Evandro Chagas National Institute of Infectious Diseases, Oswaldo Cruz Foundation, Rio de Janeiro 21040-900, Brazil; marina.furtado@ini.fiocruz.br (M.C.F.);; 3 Leonidas and Maria Deane Institute, Fiocruz, Manaus 69057-070, Brazil

**Keywords:** canine distemper virus, yellow fever virus, phylogeny, neotropical primates, mortality, coinfection

## Abstract

We describe a natural coinfection with canine distemper virus (CDV) and yellow fever virus in a free-ranging neotropical primate of the genus *Callithrix*, found dead in the northeastern region of Brazil. The laboratory diagnosis included histopathology, immunohistochemistry, rRT-PCR, and phylogenetic analyses. The CDV sequences from this primate in Brazil represent a divergent lineage in Rio Grande do Norte, closely related to genotypes EU1/South America 1 and South America 2. To our knowledge, this is the first report of natural coinfection by CDV and yellow fever virus in a neotropical primate, underscoring the need to further investigate the circulation of this virus in Brazilian nonhuman primates and its potential implications for wildlife conservation.

## 1. Introduction

The canine distemper virus (CDV) (species *Morbillivirus canis*), belonging to the genus *Morbillivirus* of the family Paramyxoviridae, is one of the major pathogens of domestic dogs and wild carnivores worldwide [1]. Several species of mammals belonging to families within the orders Carnivora (*Canidae*, *Procyonidae*, *Mustelidae*, *Ursidae*, *Viverridae*, *Felidae*, *Ailruidae*, *Hyaenidae*, *Mephitidae*, *Phocidae*, and *Otariidae*), Rodentia (*Cricetidae* and *Sciuridae*), Pilosa (*Myrmecophagidae*), Artiodactyla (*Tayassuidae*), Lagomorpha (*Leporidae*), Proboscidea (*Elephantidae*), and Primates (*Cercopithecidae*) have described CDV infection [1,2,3].

The CDV is an enveloped, non-segmented, single-stranded, negative-sense RNA virus that encodes six structural proteins. As morbilliviruses are considered the most infectious viruses known, with high morbidity and mortality rates (90–95%) in susceptible populations, it is considered a “crowd” disease, requiring populations of interacting animals to maintain the virus in a stable enzootic state [4]. The host cells that CDV uses to gain entry are SLAM (signaling lymphocyte activation molecule, CD150) and nectin-4 on epithelial cells. CDV’s ability to bind and efficiently use the SLAM receptor of many different host species makes it promiscuous [5]. CDV protein H determines the cellular tropism and host range when interacting with the SLAM receptor in host lymphoid tissues and with nectin-4 in epithelial tissues [2]. Mutations in the hemagglutinin (H) can increase species crossing [5].

Clinical manifestations of CDV infections vary widely and depend on factors such as individual immune status, vaccination history, and the CDV field strain’s virulence. The main clinical symptoms include dermatological, respiratory, gastrointestinal, neurological, and immunological disorders such as immunosuppression [6,7]. Domestic dogs have historically been considered CDV reservoirs, and they have been suggested to be the most common source of CDV transmission to wildlife. The big effect of CDV can be seen in the way it affects endangered species like Siberian tigers, Ethiopian wolves, and red pandas, as well as other weak animal species like cheetahs and lions [5,8]. With increasing research, the list of wildlife species continues to grow, and CDV is now an emerging viral risk for multiple wildlife species including Old World monkeys [8,9]. Some studies have also reported CDV infection in the family *Cercopithecidae* (Old World monkeys), with the first cases of natural CDV infection in Japanese macaques (*Macaca fuscata*) in a laboratory animal facility in 1989 [9]. In China, large outbreaks of CDV occurred in rhesus monkeys (*Macaca mulatta*) at a breeding farm in Guangxi Province in 2006, with mortality rates of up to 30% [10] and in rhesus monkeys at an animal center in Beijing in 2008 [11]. In 2008, a subsequent CDV outbreak in Japan also observed CDV in colonies of Cynomolgus macaques (*Macaca fascicularis)* [12]. To date, there have been no records of CDV in NHPs in the Americas.

Yellow fever virus (YFV), a single-stranded positive-sense RNA virus prototype of the genus *Flavivirus* of the family *Flaviviridae*, is the etiologic agent of yellow fever disease (YFD). YFV is transmitted by arthropod vectors to the human population and to nonhuman primates (NHP). In Brazil, the disease has two distinct epidemiologic transmission cycles: the sylvatic (or jungle) cycle and the urban cycle. In the sylvatic cycle, wild mosquitoes of the genera *Haemagogus and Sabethes*, along with NHP, serve as the main reservoirs of the YF virus, maintaining the virus in nature. Some genera of NHP are highly susceptible to yellow fever (YF) and can die acutely from this disease, thus playing an essential role as sentinels for YF surveillance. The disease in the urban cycle is anthroponotic, and its main vector is *Aedes aegypti*. Since 1942, due to mass vaccination, vector control programs of *Aedes aegypti* mosquitoes, and improved surveillance, the records of yellow fever cases have been exclusively associated with the sylvatic cycle [13].

A close phylogenetic relationship between nonhuman primates (NHPs) and humans is recognized as a causal factor underlying the sharing and co-evolution of zoonotic pathogens. The continued expansion of the human frontier into the wild and the urbanization of the landscape are creating interfaces where neotropical NHPs are forced to live in closer proximity to humans and domestic animals. These changes, and the interactions and dynamics between NHPs and pathogens, may increase opportunities for the spread of parasites between humans and NHPs, with implications for human and animal health and conservation [14].

In 1999, the Brazilian Ministry of Health established the NHP Epizootic Events Surveillance System to issue alerts on the risk of a YF outbreak. In recent decades, due to the outbreaks of YF in Brazil, the Brazilian National Yellow Fever Control Program has increased surveillance of deaths among free-ranging NHPs [15].

During a routine laboratory surveillance for yellow fever in a deceased NHP of the genus *Callithrix* from northeastern Brazil, YFV and CDV were detected in the animal’s tissue specimen. To our knowledge, this is the first report of YFV and CDV coinfection in a neotropical primate. This report presents histopathologic, immunohistochemical, and molecular findings, including CDV phylogenetic analysis, supporting the coinfection of these two viruses.

## 2. Materials and Methods

### 2.1. Samples

The Laboratory of Arboviruses and Hemorrhagic Viruses (LARBOH) and the Anatomic Pathology Laboratory of the Oswaldo Cruz Foundation (Fiocruz) tested fresh and formalin-fixed samples of liver, kidney, spleen, brain, and heart from a free-ranging *Callithrix* found dead in the state of Rio Grande do Norte, Brazil, in 2022, as part of YF surveillance. The fresh samples were subjected to real-time RT-PCR (rRT-PCR), and formalin-fixed samples were prepared for the histopathological and immunohistochemical investigation of YF.

### 2.2. Molecular Analyses

The MagMAX^TM^ Pathogen RNA/DNA kit (Life Technologies, Carlsbad, CA, USA) and the Kingfisher Flex Automatic Extractor (Thermo Fisher Scientific, Waltham, MA, USA) were used to obtain RNA from fresh liver, spleen, and brain fragments. The kit’s instructions were followed exactly. YFV RNA was detected in the spleen and the liver, but not in the brain, using the reverse transcription quantitative polymerase chain reaction (rRT-PCR) protocol described by PAHO [16] adapted by Domingo et al. [17], which targets the highly conserved 5′ noncoding region (5′ NC).

### 2.3. Sequencing and Genome Retrieval

To determine the genotype of YFV specimen with a positive YFV RT-qPCR result (defined as a cycle threshold (Ct) value ≤38) [16,17], Next-Generation Sequencing (NGS) was performed using the Illumina Viral Surveillance Panel (VSP), following the manufacturer’s instructions. A paired-end run (2 × 75) was conducted on the Illumina MiSeq platform at ILMD-Fiocruz Amazônia, which is part of the Fiocruz Genomics Surveillance Network. The FASTQ reads obtained for each library were trimmed for quality and potential remaining Illumina adapters using the BBDUK plugin embedded in Geneious Prime 2023.1.2. After that, the FASTQ reads were classified into taxonomic groups using the Kaiju software 1.7.3 [18] and the NCBI virus RefSeq database (14 March 2023), which can be found at https://bioinformatics-center.github.io/kaiju/downloads.html (accessed on 14 March 2023). The reads did not cluster with the YFV genome, which could be expected given the low viral load present in the specimens. However, Kaiju results showed the presence of the CDV. Therefore, we first assembled the reads against the CDV RefSeq using BBMap, also known as Bedded in Geneious Prime. The consensus sequence was used for a nucleotide BLAST search, which returned the GenBank record MH349541 as the closest match. Thus, we performed a second assembly using this record as a template, and the final consensus showed 97% identity with the CDV genome. Accordingly, we used this assembled genome for phylogenetic analysis. Concurrently, we employed a de novo assembly strategy using the SPAdes assembler 3.15.5 software (https://currentprotocols.onlinelibrary.wiley.com/doi/abs/10.1002/cpbi.102, accessed on 14 March 2023), which generated a sequence near 11 kb that we later identified as CDV by using a nucleotide BLAST search against the entire non-redundant GenBank database. Thus, two independent methods recovered the CDV genome in the investigated specimens.

### 2.4. Phylogenetic Analyses

The final datasets for phylogenetic analysis contained 591 H gene sequences, while 30 sequences were used for the genomes. For the analysis of pairwise distances, we used sequences from the H gene. All complete gene H sequences (1800 bp) deposited in GenBank were selected, which contained information on the year of collection, country of isolation, and host. This resulted in a final dataset of 591 H sequences that were aligned using the MAFFT version 7 program [19]. Pairwise distance matrices between strains were calculated using the MEGA 11 version 11.0.13 software [20] and included 19 distinct strains present in different parts of the world [21].

A comprehensive whole-genome analysis was conducted on 17 strains, in addition to two vaccine-related strains (Rockborn-like and Onderstepoort vaccine). The sequences available at the GenBank were spread across genotypes: Europe/South America 1, South America 2, South America 2 Argentina, South America 3, South America 4/North America 4, America 2, Asia 1, Asia 2, Asia 4, Asia 6, Asia 5, Africa 1 (South Africa), Africa 2 (Tanzania), Arctic/Arctic-like, America 1 (vaccine like), Caspio, and Australia. Some strains were excluded from the study due to the partial nature of the North America 3, America 5, Asia 3, and Europe Wildlife strains, which only encompass a portion of the H gene sequence. For whole-genome analysis, the initial datasets for pairwise distance analysis contained 102 full-genome sequences. Multiple sequence alignments were generated using the online service of the MAFFT version 7 program [19] with default parameters. We carried out phylogenetic reconstruction using IQtree server version 1.6.12 [22], performing 1000 replicates of ultrafast bootstrapping [23] under the GTR+F+I+G4 substitution model, as suggested by ModelFinder [24]. We then edited the final tree using FigTree version 1.4.4.

Sequences with more than 98% similarity were excluded based on genotypic patterns, and 33 genomes of CDV were analyzed and 17 previously characterized strains were included. The phocine distemper virus (PDV/Wadden Sea. NLD/1988 COI) (GenBank accession number: NC028249) was employed as an outgroup for the purpose of facilitating enhanced visualization of the tree and was trimmed accordingly.

Of the 33 selected sequences, 30 were used after removing three potentially recombinant genomes (GenBank accession number: JX681125.1, MT012803.1, and KJ994343.1), as shown by the RDP5 v5.5 software [25].

Sequences were aligned with the NHP sequence using the MAFFT program from the Geneious Prime^®^ 2023.2.1 software package with default parameters. We conducted Maximum Likelihood (ML) phylogenetic analysis using IQtree server version 1.6.12 [22], performing 1000 replicates of ultrafast bootstrapping [23] under the GTR+F+I+G4 substitution model, as indicated by ModelFinder [24], and visualizing the final tree in FigTree version 1.4.4. Additionally, since most available CDV sequences are partial, we reconstructed an ML phylogenetic tree with 590 complete H sequences from around the world. In addition to the NHP sequences, 591 sequences distributed in 19 genotypes were analyzed: Europe/South America 1, South America 2, South America 2 Argentina, South America 3, South America/North America 4, America 1, America 2, North America 3, Asia 1, Asia 2, Asia 4, Asia 5, Asia 6, Africa 1 (South Africa), Africa 2 (Tanzania), Europe wildlife, Artic/Artic-like, Australia, and Caspio. Sequences were analyzed with NHP using the methods described above.

### 2.5. Histopathological and Immunohistochemical Analyses

Formalin-fixed paraffin-embedded (FFPE) samples of liver, lung, spleen, kidney, brain, and heart tissues were sectioned at a thickness of 5 μm for routine histological staining with hematoxylin and eosin (H&E).

For immunohistochemistry (IHC), 4 μm tissue sections were placed onto silanized slides that were dewaxed and rehydrated through xylene and alcohol, respectively. The sections underwent antigen retrieval with Reveal Decloacker RTU (Biocare Medical^®,^, Pacheco, CA, USA) in a digital electrical pressure cooker. The slides were incubated overnight with anti-YFV polyclonal antibody (Virology Center, Adolfo Lutz Institute) or with anti-CDV monoclonal antibody (Invitrogen^®^ MA1-82327, clone DV2-12, Carlsbad, CA, USA), followed by MACH 4 Universal AP-Probe (Biocare Medical^®^, Pacheco, CA, USA) and MACH 4 Universal AP-Polymer (Biocare Medical^®^). The reactions were visualized by using fast red chromogen Warp Red Chromogen Kit (Biocare Medical^®^, Pacheco, CA, USA) and the slides were counterstained with Harris hematoxylin. As a positive control, samples from an NHP naturally infected with YFV and from a dog naturally infected with CDV were used.

## 3. Results and Discussion

The animal described in this study was a wild animal found dead during passive surveillance of YFV in the state of Rio Grande do Norte, in the northeastern region of Brazil. The spleen and liver samples had YFV RNA detected by rRT-PCR. The spleen had a viral load of 36, and the liver had a viral load of 38, as shown by a cycle threshold (Ct) value.

**Figure 1:**
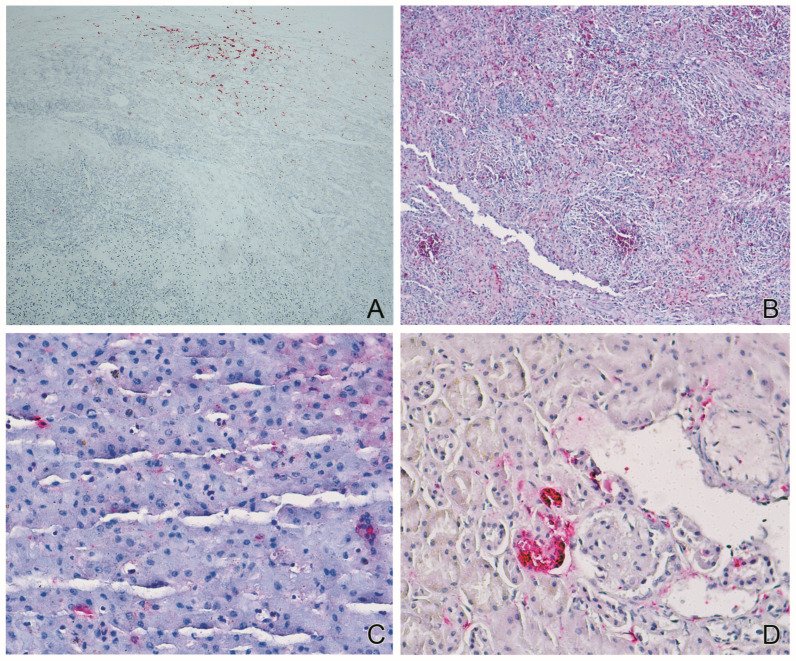
Yellow fever virus (YFV) and canine distemper virus (CDV) immunohistochemistry (IHC) in tissues from a free-ranging *Callithrix* primate. An alkaline phosphatase polymer system with fast red chromogen. (**A**) Focal granular immunostaining of YFV antigens in a necrotic area of the spleen (IHC, original magnification ×100). (**B**) Widespread immunostaining of CDV antigens in the spleen (IHC, original magnification ×100). Immunostaining of CDV antigens in the liver (**C**) and kidney (**D**) (IHC, original magnification ×400).

The IHC yielded a positive result for YFV solely in the spleen sample (Figure 1A). The histopathology of the spleen showed necrotic splenitis, lymphoid depletion, presence of multinucleated cells, and nuclear and cytoplasmic eosinophilic inclusion bodies (IBs) in lymphoid cells. The YFV-IHC showed focal granular positive immunostaining on a necrotic area of the spleen tissue (Figure 1A). The liver showed periportal lymphoplasmacytic infiltrate, multifocal necrosis, sinusoidal leukocytosis, multinucleated hepatocytes, as well as nuclear and cytoplasmic IBs in hepatocytes and cytoplasmic IBs in bile duct epithelial cells.

Additionally, the histopathology showed non-suppurative encephalitis, interstitial nephritis, and interstitial pneumonia with syncytial cells. All assessed organs had lymphoplasmacytic inflammation and necrosis. There were IBs in glia cells, cardiomyocytes, renal tubular epithelial cells, bronchial and bronchiolar epithelial cells, and alveolar septal cells. Histopathological changes compatible with YF were also observed in this case, such as hepatic necrosis, interstitial nephritis, and splenic lymphoid depletion of Malpighian corpuscles [26]. However, significant changes, such as pneumonia, encephalitis, the presence of multinucleated cells, and eosinophilic IBs, suggested the involvement of another virus besides YFV.

Nevertheless, genome sequencing of the YFV was not generated due to the low viral load in the samples. The presence of YFV antigen in the spleen, as detected by IHC, ratified the rRT-PCR findings.

To genotype the YFV, the samples amplified by rRT-PCR were analyzed using an unbiased Next-Generation Sequencing (NGS) approach that revealed the presence of the CDV genome. We used a commercial panel for nucleotide sequencing based on hybrid capture enrichment (Illumina Viral Surveillance Panel). This kit uses probes designed to capture 66 viruses identified as high-risk to public health, including the YFV. However, since several probes are included for each prototype virus, Illumina’s internal data show that the VSP kit may also successfully amplify and sequence other similar viruses. Given that CDV is a paramyxovirus, like the respiratory syncytial virus and influenza virus included in this kit, it is not surprising that VSP also detects other members of this family based on sequence similarities.

Another critical point that allowed us to identify CDV was the two bioinformatic strategies employed to analyze the data generated in our sequencing run. First, we applied Kaiju, a sensitive taxonomic classification tool for metagenomics data [18], directly to the FASTQ reads. Thus, Kaiju’s results identified CDV among the identified reads. Secondly, we also used an unbiased de novo assembly strategy using the SPAdes assembler 3.15.5 software. Using this method, we were able to find a sequence of about 11 kb that we later identified as CDV by using a nucleotide BLAST. The complete genome sequence of this CDV strain was obtained and submitted to the GenBank database (accession number: PQ464015).

With the results obtained by NGS, we observed that the histopathologic findings were consistent with canine distemper described in domestic dogs and various wild species [9,12,27,28]. We then performed IHC, which detected CDV antigens (Figure 1B–D) in the tissues of the liver, lung, spleen, kidney, brain, and heart.

**Figure 2:**
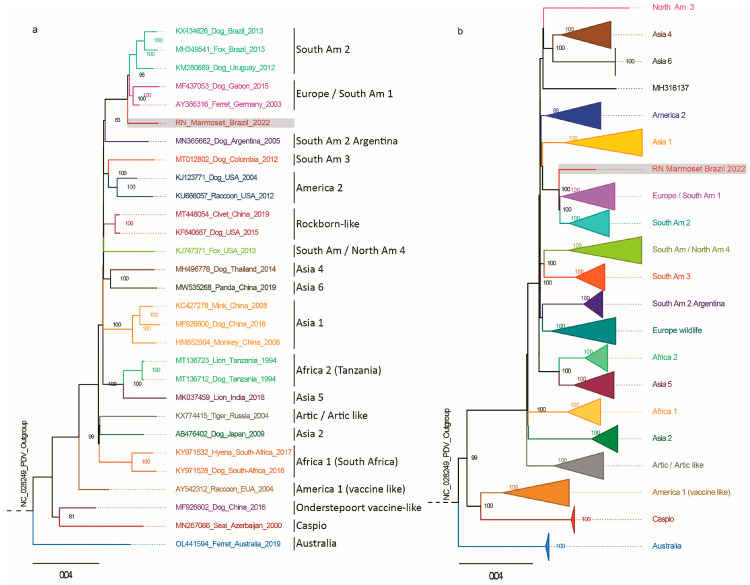
Phylogenetic analysis was performed using the genome of the CDV strain isolated from a neotropical primate in Brazil. (**a**): Maximum likelihood (ML) phylogenetic analysis was performed on the genome using a data set of complete nucleotide sequences (N = 30). (**b**): Maximum likelihood (ML) phylogenetic analysis was generated for the H gene (N = 591) using sequences with a total length of 1800 bp. The sequence from the Rio Grande do Norte animal is highlighted in the grey box. The phocine distemper virus was used as an outgroup.

The neotropical primate genome contained a structure and sequence typical of CDV encoding the predicted structural genes, similar to that found in domestic dogs’ viruses. The phylogenetic location of CDV in neotropical primates was determined using a dataset that contained all the full genomes available in GenBank. Using the sequence from this study and the 29 full representative downloads from GenBank, we made an ML phylogenetic tree with 30 complete genomic sequences (Figure 2a). Since most available CDV sequences are partial, we reconstructed an ML phylogenetic tree with 591 complete H sequences from around the world (Figure 2b). In both phylogenetic analyses, the CDV sequence of NHP clustered with high support (100 bootstraps) basal to the genotypes Europe/South America 1 and South America 2. Thus, the CDV sequences isolated from this primate in Brazil represent a divergent lineage in Rio Grande do Norte, closely related to the genotypes Europe/South America 1 and South America 2.

A distance matrix was constructed using the 590 complete H gene sequences available worldwide, due to the widespread use of the H gene for lineage demarcation. The results of the analysis indicated that the mean genetic distance between the 19 CDV lineages ranged from 4.4 to 13% at the amino acid level (aa) (Table 1). The sample collected in Rio Grande do Norte exhibited a lower average genetic divergence of amino acids for South America 2 (*p* 3.8% aa), thereby substantiating its classification as a member of this lineage in accordance with the criteria for CDV lineage classification established by other authors. In accordance with the prevailing methodology for CDV lineage classification based on the H gene sequence, a novel strain is deemed a distinct lineage if it exhibits a minimum of 5% nucleotide divergence or 4% amino acid divergence from existing lineages [21,29,30,31,32,33].

Although some *Callithrix* individuals may present severe consequences of YF, such as fatal hepatic failure, this genus seems to be more resistant to YFV infection when compared to other genera. *Callithrix* commonly presents a lower YFV viral load, and may show no significant hepatic lesions and no evident antigen in IHC, despite detectable viral RNA [34]. The genetic evidence, along with the extensive presence of CDV antigens and severe histopathological changes consistent with canine distemper, strongly suggests CDV as the primary cause of death. Despite low-level YFV detection in the spleen and liver, its impact on the outcome is likely negligible; however, the combined presence of YFV and CDV’s immunosuppressive effects may also have contributed to the animal’s morbidity [35].

Few cases of natural CDV infection in NHPs have been previously described in Old World captive animals [10,11,12,27]. Extensive reports of CDV in various wildlife species worldwide, affecting both wild carnivores and non-carnivores, have been documented [2,5]. In Brazil, several reports have documented CDV in a hoary fox (*Lycalopex vetulus*), a lesser grison (*Galictis quem*), coatis (*Nasua nasua*), and felids (*Puma concolor*, *Leopardus wiedii*, *and Herpailurus yagouaroundi*) [36], and in a lesser anteater (*Tamandua tetradactyla*) [37]; however, to date, there have been no records of CDV in NHPs.

Neotropical primates are arboreal, and the habitat degradation through deforestation is forcing them to use different elevation levels; these conditions may trigger opportunities for shedding infectious agents to other individuals and species, including increased contact with pathogens transmitted indirectly (e.g., orofecally, through bodily fluids or virus-contaminated food, etc.) that are not distributed in the tree canopy [14].

Considering the intricate interactions between nonhuman primates (NHPs), wild animals, human populations, and domestic animals, particularly involving primates of the genus *Callithrix*, which demonstrate a capacity to transition between different environments [38,39], it is challenging to ascertain the association between exposure rates. This is because most studies on CDV risk factors concentrate on individual animal characteristics, while the impact of environmental factors is likely linked to the complex movement patterns of this animal species [36].

## 4. Conclusions

Although we have detected the presence of YFV through IHC and rRT-PCR analyses, the incidental discovery of CDV through metagenomics, combined with widespread CDV antigens and severe histopathological changes consistent with canine distemper, strongly suggests CDV as the primary cause of death in this case. These findings together demonstrate the importance of a robust laboratory investigation.

The discovery of CDV in naturally infected neotropical primates leads us to consider the epidemiologic relationship between these animals and the potential for viral persistence in urban and wild environments. Therefore, further investigation into the circulation of CDV among neotropical primates and increased wildlife surveillance are critical to understanding and identifying potential sources of infection in these animals. In addition, increasing knowledge of the distribution and interrelationships of CDV and possible meta-reservoirs underscore the importance of comprehensive research to monitor the potential for species jumps and mitigate associated risks, in addition to conservation efforts for primate species.

## Figures and Tables

**Table 1:** Mean amino acid distance matrix of the translated H gene among CDV lineages (*p* %).

		1	2	3	4	5	6	7	8	9	10	11	12	13	14	15	16	17	18	19	20
1	Australia																				
2	Caspio	10.6																			
3	America 1	8.9	8.8																		
4	North Am 3	11.1	13.0	10.2																	
5	Asia 1	10.5	11.6	9.5	8.1																
6	South Am 2	9.8	10.7	8.6	6.9	6.1															
7	Europe/South Am 1	9.7	11.1	9.5	7.4	6.3	4.4														
8	RN Marmoset Brazil	10.1	10.9	9.1	7.1	6.3	3.8	4.6													
9	America 2	10.5	11.1	9.1	7.6	6.3	5.3	5.8	5.5												
10	Europe wildlife	10.4	11.6	9.4	7.3	6.6	5.9	6.0	6.0	6.0											
11	South Am 3	10.9	11.4	9.7	7.5	6.8	5.6	6.1	5.4	6.1	6.5										
12	South Am/North Am 4	10.7	12.1	9.7	7.9	7.1	6.1	6.5	6.4	6.5	6.4	6.1									
13	South Am 2 Arg	10.5	10.3	9.0	7.0	6.4	4.8	5.3	4.8	5.6	5.3	5.9	6.1								
14	Asia 4	10.3	11.5	9.0	6.9	6.0	5.5	5.7	5.3	5.8	5.8	5.5	5.7	5.3							
15	Africa 2	10.5	11.1	8.9	7.5	6.4	6.0	6.2	5.9	6.0	5.9	6.8	6.8	6.1	6.0						
16	Asia 5	11.6	12.0	9.4	8.1	7.5	6.5	7.2	6.8	7.1	7.5	7.6	7.9	7.4	7.4	5.3					
17	Artic/Artic like	11.0	11.6	9.8	8.9	7.9	7.1	7.7	7.1	7.6	7.5	7.9	7.7	7.4	7.4	7.7	8.9				
18	Africa 1	9.2	11.1	8.7	7.7	6.2	5.9	6.5	6.0	6.2	6.3	5.9	6.5	6.0	6.0	6.1	7.6	6.4			
19	Asia 6	12.3	12.7	10.6	8.9	7.5	7.2	7.6	7.1	7.2	7.5	7.5	7.7	7.1	5.7	7.8	9.1	9.1	7.7		
20	Asia 2	11.5	12.5	10.2	8.5	7.5	7.5	7.8	8.0	7.9	8.3	7.9	8.1	7.8	7.8	8.1	8.8	8.4	7.0	9.3	

Notes: South Am 2: South America 2; Europe/South Am 1: Europe/South America 1; South Am 2 Arg: South America 2 (Argentina); South Am/North Am 4: South America/North America 4; South Am 3: South America 3.

## Data Availability

All data necessary to evaluate the conclusions of the paper are included in the paper and the new sequence generated as part of this study has been deposited in GenBank under accession number PQ464015.
